# Differences in cerebral cortical anatomy of left- and right-handers

**DOI:** 10.3389/fpsyg.2014.00261

**Published:** 2014-03-28

**Authors:** Tulio Guadalupe, Roel M. Willems, Marcel P. Zwiers, Alejandro Arias Vasquez, Martine Hoogman, Peter Hagoort, Guillen Fernandez, Jan Buitelaar, Barbara Franke, Simon E. Fisher, Clyde Francks

**Affiliations:** ^1^Language and Genetics Department, Max Planck Institute for PsycholinguisticsNijmegen, Netherlands; ^2^International Max Planck Research School for Language SciencesNijmegen, Netherlands; ^3^Centre for Cognitive Neuroimaging, Donders Institute for Brain, Cognition and Behaviour, Radboud University NijmegenNijmegen, Netherlands; ^4^Neurobiology of Language Department, Max Planck Institute for PsycholinguisticsNijmegen, Netherlands; ^5^Department of Human Genetics, Radboud University Medical CenterNijmegen, Netherlands; ^6^Department of Psychiatry, Donders Institute for Brain, Cognition and Behaviour, Radboud University Medical CenterNijmegen, Netherlands; ^7^Department of Cognitive Neuroscience, Donders Institute for Brain, Cognition and Behaviour, Radboud University Medical CenterNijmegen, Netherlands; ^8^Donders Institute for Brain, Cognition and Behavior, Radboud University NijmegenNijmegen, Netherlands

**Keywords:** MRI, handedness, cortical surface area, brain asymmetry, FreeSurfer

## Abstract

The left and right sides of the human brain are specialized for different kinds of information processing, and much of our cognition is lateralized to an extent toward one side or the other. Handedness is a reflection of nervous system lateralization. Roughly ten percent of people are mixed- or left-handed, and they show an elevated rate of reductions or reversals of some cerebral functional asymmetries compared to right-handers. Brain anatomical correlates of left-handedness have also been suggested. However, the relationships of left-handedness to brain structure and function remain far from clear. We carried out a comprehensive analysis of cortical surface area differences between 106 left-handed subjects and 1960 right-handed subjects, measured using an automated method of regional parcellation (FreeSurfer, Destrieux atlas). This is the largest study sample that has so far been used in relation to this issue. No individual cortical region showed an association with left-handedness that survived statistical correction for multiple testing, although there was a nominally significant association with the surface area of a previously implicated region: the left precentral sulcus. Identifying brain structural correlates of handedness may prove useful for genetic studies of cerebral asymmetries, as well as providing new avenues for the study of relations between handedness, cerebral lateralization and cognition.

## Introduction

Handedness is perhaps the most overt reflection of lateralization of the central nervous system in humans. Humans show a strong and population-level bias toward using one hand rather than the other for manual activities, which is unusual among mammals (Vallortigara et al., 2011). Roughly 90% of humans are right-handed, while even other primates (e.g., chimpanzees and macaques) do not show such a strong degree of population-level handedness (Lonsdorf and Hopkins, 2005; Meunier et al., 2013). This motor asymmetry is observable at least as early during human development as 15 weeks of gestation, and is preceded by asymmetries of arm movements even earlier (Hepper, 2013). In addition the tendency toward right handedness has apparently been present throughout human history, and across cultures and continents (Coren and Porac, 1977; Hardyck and Petrinovich, 1977; McManus, 1991, 2009; Faurie and Raymond, 2004).

Due in part perhaps to its minority status and past cultural stigmatization, left-handedness has often been studied in the context of pathology, for example in relation to Alzheimer's disease (de Leon et al., 1986), substance use (London, 1989), and autoimmune disorders (Geschwind and Behan, 1982). Handedness has also been investigated in relationship to lateralized cognitive functions, such as visuospatial processing (Gordon and Kravetz, 1991), face recognition (Luh et al., 1994; Willems et al., 2010; Bukowski et al., 2013) and prominently, language (Tzourio et al., 1998; Knecht et al., 2000b). Knecht and colleagues found an increased incidence of bilateral and right hemisphere language lateralization among left-handers, compared to right-handers, although the majority of left/mixed handers still showed left-hemisphere language dominance (Knecht et al., 2000a,b). This suggests that developmental mechanisms affecting cerebral language dominance overlap to an extent with those influencing hand motor control. However, it remains poorly understood how these different domains of functional lateralization are related to each other (Badzakova-Trajkov et al., 2010).

Several early attempts to understand human handedness attributed right-handedness to socio-cultural, anatomical, as well as genetic factors (for a review see Hardyck and Petrinovich, 1977 or Corballis et al., 2012 for a more recent one). However, the developmental basis of human brain lateralization remains almost wholly unknown, and likewise the causes of its variation are hardly understood (Willems et al., 2014). One robust observation is that males show a slightly higher proportion of left-handedness than females (Halpern et al., 1998; Peters et al., 2006; Sommer et al., 2008). Recent twin studies, based on thousands of families, have indicated that 21–24% of the liability to left- handedness can be explained by additive genetic effects (Medland et al., 2009; Vuoksimaa et al., 2009). This indicates that genetic variation plays a role in causing variation in handedness. In contrast to original models of handedness as a monogenic trait (Annett, 1985; McManus, 1985), recent evidence from genome-wide association studies strongly suggest more complex models (Medland, 2009; McManus et al., 2013; Armour et al., 2014). So far, studies aimed at discovering the specific genetic loci involved have yielded tentative associations with the genes AR, APOE, COMT, PCSK6, LRRTM1 (Medland et al., 2005; Francks et al., 2007; Savitz et al., 2007; Bloss et al., 2010; Scerri et al., 2011; Brandler et al., 2013). Although originally discovered in populations affected by dyslexia, PCSK6 has also shown association with degree of handedness in a healthy sample of unrelated adults (Arning et al., 2013). It is not yet known how these genes may influence asymmetrical development of the brain (see Ocklenburg et al., 2013).

Identifying brain anatomical correlates of left-handedness may provide potential endophenotypes for further genetic association studies (Ocklenburg et al., 2013; Willems et al., 2014). Finding anatomical correlates of left-handedness may also inform on the relations between handedness and lateralized cognitive functions, and more broadly on brain structure-function relationships (Ocklenburg et al., 2013; Willems et al., 2014). Amunts et al. (1996) found deeper left precentral sulci in right-handers than left-handers using manual segmentations of magnetic resonance (MR) images. Consistent with this, Foundas et al. (1998) examined left-right asymmetries of the precentral gyrus in a sample of 15 left- and 15 right handers based on manual segmentations of their MR images, and found leftward asymmetries in right-handers, but no consistent asymmetry in left-handers (also see Kloppel et al., 2007 and Willems and Hagoort, 2009, for corroborating findings using functional MR imaging). More recently, gray matter volume in the central sulcus was shown to relate to hand motor skill, but to different extents depending on handedness (Herve et al., 2005). In addition, asymmetry of the planum temporale (PT), the posterior portion of the superior surface of the temporal lobe, has been reported to associate with hand preference (Steinmetz et al., 1991; Foundas et al., 1995; Herve et al., 2006). However, results regarding the PT have not been consistent throughout the literature (Witelson and Kigar, 1992; Good et al., 2001). Similarly, an association between handedness and cerebral torque, another structural brain asymmetry, has also been assessed with inconclusive results (Narr et al., 2007). More recently, Powell et al. (2012) in a study of 40 left-handers and 42 right-handers found differences in sulcal shape of the pars orbitalis (PO) and pars triangularis (PTr), as well as differences of volumetric asymmetry within the PO. To our knowledge, Good et al. (2001) has studied the largest sample to have been used in examining brain morphological differences related to handedness. Using a voxel-based morphometry analysis with a total sample of 465 subjects (67 lefthanders) they did not find structural correlates of handedness in the brain. This suggests that any such correlates are subtle and will require larger samples and/or other ways to quantify brain structure, in order to detect them unambiguously.

The goal of the present study was to identify cerebral cortical differences between left and right-handers, by analyzing the largest sample used so far for this purpose (106 left-handed subjects and 1960 right-handed subjects), and using recently developed methodology for the automated segmentation and quantification of regional gray matter (Fischl et al., 2004). We analyzed the data in three stages. First we examined total cortical surface area in relation to handedness. Then, we tested a set of candidate cortical regions for associations with handedness, based on the previous studies mentioned above. Finally, we carried out a screen over all remaining cortical regions.

## Methods

### Study dataset

The Brain Imaging Genetics (BIG) study was initiated in 2007 and comprises healthy volunteer subjects, including many university students, who participate in diverse imaging studies at the Donders Centre for Cognitive Neuroimaging (DCCN), Nijmegen, The Netherlands (Franke et al., 2010). At the time of this study the BIG subject-pool consisted of 2337 self-reported healthy individuals (1248 females) who had undergone anatomical (T1-weighted) MRI scans, usually as part of their involvement in diverse smaller-scale studies at the DCCN, and who had given their consent to participate in BIG. Their median age was 23 years. A subset of 235 subjects had undergone a brain MRI scan twice, with at least 1 day separation between scans. Fifty percent of the 235 re-scans took place within 181 days of the first, with the mean elapsed time being 320 days (*SD* = 360). At the time of the first scan, the median age of this group was 23 years.

Handedness of the participants was assessed by an item in their enrolment form. This consisted of subjects selecting the appropriate label, either “left-handed/right-handed” (in Dutch). We discuss the validity of this method of assessing handedness further below. Only those subjects who clearly indicated one or the other state were included in our analysis. This resulted in a sample of 1960 right-handed subjects and 106 left-handed subjects, with a median age of 22 years and a standard deviation of 11 years. The proportion of left-handers was substantially lower than in the general population; this was due to left- handedness being used as an exclusion criterion for some of the imaging studies that were pooled into the overall BIG dataset.

### Image acquisition

MRI data in BIG were acquired with either a 1.5 Tesla Siemens Sonata or Avanto scanner or a 3 Tesla Siemens Trio or TimTrio scanner (Siemens Medical Systems, Erlangen, Germany). Given that images were acquired during several smaller scale studies, the parameters used were slight variations of a standard T1-weighted three-dimensional magnetization prepared rapid gradient echo sequence (MPRAGE; 1.0 × 1.0 × 1.0 mm voxel size). The most common variations in the TR/TI/TE/sagittal-slices parameters were the following: 2300/1100/3.03/192, 2730/1000/2.95/176, 2250/850/2.95/176, 2250/850/3.93/176, 2250/850/3.68/176, 2300/1100/3.03/192, 2300/1100/2.92/192, 2300/1100/2.96/192, 2300/1100/2.99/192, 1940/1100/3.93/176 and 1960/1100/4.58/176. There was also variation in the number of headcoils used across BIG scans, however, no systematic differences were observed in their use between left- and right-handed subjects. The following arrays were employed (and their frequencies) in the right-handed population: 32-channel (24%), 12-channel (4%), 8-channel (38%), arrays and single headcoil (33%). In the left-handed population, this distribution was 32-channel (27%), 12-channel (0%), 8-channel (33%), arrays and single headcoil (40%).

### Image processing

Automated parcellation of cerebral cortical regions from T1-weighted images was done in FreeSurfer v5.1 (Fischl et al., 2004) according to the Destrieux atlas (Destrieux et al., 2010) within the “-recon-all” processing pipeline, and using default parameters. Measures of surface area (in mm^2^) were produced for the total cortical surface and for each of 74 cortical parcellations, in each hemisphere. Outlier values (more extreme than 3.5 *SD* from the mean) were excluded for each measure. The scan–rescan correlation of each measure was then calculated in the sample of 235 subjects who had undergone two MRI scans, after correcting for the potential covariate effects of age, sex, total cortical surface area and scanner field strength (IBM SPSS v.20).

Out of the 74 covariate-corrected bilateral cortical measures, 23 were excluded from subsequent analyses, due to low scan–rescan correlation in either left, right or both structures (Pearson's *r* < 0.7; i.e., corresponding to shared proportion of variance between scan and re-scan measures of <0.49). Regional measures of cortical thickness were also generated. There is evidence that cortical surface and thickness have independent sources of variation (Panizzon et al., 2009). However, we discarded the thickness measures because the majority (81%) showed scan–rescan correlations below 0.7.

### Cortical correlates of handedness

We tested for associations between handedness and cortical surface areas using repeated-measures ANOVA, implemented in SPSS (IBM SPSS v.20). Hemisphere (left vs. right) was factored as a within-subjects variable and handedness group as a between-subjects variable in a full factorial design. This allowed the detection of bilateral associations of handedness with cortical surface areas, as well as asymmetrical associations (by means of the interaction between handedness and hemisphere). We first tested the total hemispheric surface areas, and then we tested the regional surface areas. In addition, the following covariates were entered into the analyses: sex, age, scanner field strength, and total (i.e., left plus right) hemispheric surface area (the latter only for the analyses of regional surfaces).

We tested candidate cortical regions motivated by previous findings in the literature (specifically by the studies reviewed in the introduction). We separated these candidate regions into three domains; language, motor control and visual processing. Language-related candidate regions were the inferior frontal gyrus and superior temporal gyrus. These corresponded most closely to the following parcellations within the Destrieux atlas, that had also showed a robust scan–rescan correlation: Opercular part of the inferior frontal gyrus, triangular part of the inferior frontal gyrus, anterior transverse temporal gyrus (of Heschl), lateral aspect of the superior temporal gyrus, and PT. The motor control candidate regions were the superior and inferior parts of the precentral sulcus (as defined in the Destrieux atlas). The visual-related candidate regions comprised inferior and ventral areas of the temporal lobe. In the Destrieux atlas these corresponded most closely to the following regions: inferior temporal gyrus, lateral occipito-temporal gyrus (fusiform gyrus) and lingual part of the medial occipito-temporal gyrus. We applied Bonferroni corrections for the comparisons done within each of these domains.

After the analysis of candidate regions, we then tested all of the remaining cortical regions for differences between left- and right-handers, again using Bonferroni adjustment to correct for multiple testing.

### Power analysis

We used G * Power v3.1.9 (Faul et al., 2009) to estimate the necessary effect sizes to be detected given our study design. We considered our sample size, a required power (1-β) of 80%, a correlation between bilateral volumes of *r* ~0.8, and an α level corrected for multiple testing. This resulted in estimates of partial η^2^~0.07 [*F*_(1, 2055)_ ~5.7] for analyses within each of the candidate domains, and a partial η^2^~0.09 [*F*_(1, 2055)_ ~10] for the analysis of the remaining cortical surfaces. In other words we had 80% power to detect an association explaining 9% of the residual variance in a regional cortical surface area after having removed the effects of covariates and after considering the multiple comparisons, for the screening analysis of non-candidate regions.

## Results

The proportion of left-handers in our sample differed significantly between males and females. Of the 942 males, 59 were left-handed (6.3%), and of the 1077 females, 47 were left-handed (4.4%); χ^2^_(1)_ = 4.56, *p* = 0.02, phi = 0.047.

Handedness did not show a significant association with bilateral hemispheric surface area, nor with overall hemispheric surface asymmetry (see Tables 1, 2). None of the candidate regions, related to either language, visual processing, or motor control showed significant evidence for association with handedness after correction for multiple testing within each of these domains (see Table 3). The only regions showing main effects of handedness with *p* < 0.05 before correction for multiple testing were the superior precentral sulcus and the inferior temporal gyrus. Means (and *SD*s) for these regions, by hemisphere and handedness group, are shown in Table 4.

**Table 1:** **Mean surface areas (and *SD*s) for the left and right hemispheres, by handedness**.

	**Left-handers**	**Right-handers**
Left hemisphere surface area	87855.1 (7717.6)	87984.5 (8469.9)
Right hemisphere surface area	87817.2 (8133.5)	88295.6 (8487.4)

**Table 2:** **Repeated-measures ANOVA results from testing for an association between handedness and total hemispheric cortical surface areas**.

	**Repeated-measures ANOVA**		
	***P***	***F***	**Partial η**^2^****
Handedness	0.114	2.501	0.001
Handedness * Hemisphere	0.132	2.266	0.001
Sex	<0.001	1193.7	0.367
Age	<0.001	90.1	0.042
Scanner field strength	<0.001	12.48	0.006

**Table 3:** **Summarized results for the candidate cortical regions**.

		**Repeated-measures ANOVA**		
		***P***	***F***	**Partial η**^2^****
**LANGUAGE-RELATED**				
Opercular part of the interiorfrontal gyrus	Handedness	0.73	0.12	<0.001
	Handedness * Hemisphere	0.63	0.23	<0.001
Triangular part of the inferior frontal gyrus	Handedness	0.88	0.02	<0.001
	Handedness * Hemisphere	0.17	1.8	0.001
Anterior transverse temporal gyrus (of Heschl)	Handedness	0.86	0.03	<0.001
	Handedness * Hemisphere	0.06	3.4	0.002
Lateral aspect of the superior temporal gyrus	Handedness	0.57	0.33	<0.001
	Handedness * Hemisphere	0.36	0.85	<0.001
Planum temporale	Handedness	0.42	0.64	<0.001
	Handedness * Hemisphere	0.94	0.01	<0.001
**MOTOR CONTROL-RELATED**				
Superior part of the precentral sulcus	Handedness	0.044	4.07	0.002
	Handedness * Hemisphere	0.6	0.28	<0.001
Inferior part of the precentral sulcus	Handedness	0.76	0.09	<0.001
	Handedness * Hemisphere	0.85	0.03	<0.001
**VISUAL-RELATED**				
Inferior temporal gyrus	Handedness	0.037	4.36	0.002
	Handedness * Hemisphere	0.58	0.3	<0.001
Lateral occipito-temporal gyrus (fusiform gyrus)	Handedness	0.17	1.87	0.001
	Handedness * Hemisphere	0.53	0.4	<0.001
Lingual part of the medial occipito-temporal gyrus	Handedness	0.26	1.27	0.001
	Handedness * Hemisphere	0.1	2.67	0.001

Reported are p-values before correction for multiple testing (none survived this correction).

**Table 4:** **Means (and *SD*s) for the superior part of the precentral sulcus, and inferior temporal gyrus, by hemisphere and handedness group**.

	**Left hemisphere**		**Right hemisphere**	
	**Left-handers**	**Right-handers**	**Left-handers**	**Right-handers**
Superior part of the precentral sulcus	914.9 (207.5)	952.7 (200.9)	965.1 (201.9)	990.4 (214.8)
Inferior temporal gyrus	1853.2 (328.6)	1911.7 (311.3)	1744.8 (319.6)	1787.6 (281.8)

Tables 5, 6 show results for the remaining (non-candidate) regional surface areas that reached nominal significance (i.e., uncorrected *p* < 0.05) for an association with handedness, either as a main effect on bilateral surface or as an interaction with hemisphere. None of these associations survived correction for multiple testing. The results for all cortical regions and covariates, regardless of nominal significance, can be found in Supplementary Material, together with descriptive statistics of all metrics, per handedness group.

**Table 5:** **Summary results for non-candidate cortical regions that achieved nominal significance in ANOVA**.

**Cortical surface areas**		**Repeated-measures ANOVA**		
		***P***	***F***	**Partial η**^2^****
Anterior part of the cingulate gyrus and sulcus (ACC)	Handedness	0.139	2.19	0.001
	Handedness * Hemisphere	0.023	5.18	0.003
Middle-anterior part of the cingulate gyrus and sulcus (aMCC)	Handedness	0.67	0.18	<0.001
	Handedness * Hemisphere	0.003	8.99	0.005
Superior occipital gyrus (O1)	Handedness	0.04	4.23	0.002
	Handedness * Hemisphere	0.255	1.3	<0.001
Posterior transverse collateral sulcus	Handedness	0.648	0.21	<0.001
	Handedness * Hemisphere	0.048	3.92	0.002
Superior frontal sulcus	Handedness	0.038	4.31	0.002
	Handedness * Hemisphere	0.221	1.5	<0.001
Sulcus intermedius primus (of Jensen)	Handedness	0.743	0.14	<0.001
	Handedness * Hemisphere	0.037	4.37	0.002
Parieto-occipital sulcus (or fissure)	Handedness	0.029	4.8	0.002
	Handedness * Hemisphere	0.25	1.32	<0.001

None of these results survived correction for multiple testing. Complete results for all regions and covariates are in Supplementary Table 1.

**Table 6:** **Means (and *SD*s) for non-candidate cortical regions that achieved nominal significance in ANOVA, by hemisphere and handedness group**.

	**Left hemisphere**		**Right hemisphere**	
	**Left-handers**	**Right-handers**	**Left-handers**	**Right-handers**
Anterior part of the cingulate gyrus and sulcus (ACC)	1648.0 (223.2)	1707.4 (264.5)	1998.8 (251.3)	2016.7 (271.1)
Middle-anterior part of the cingulate gyrus and sulcus	974.6 (144.7)	1014.5 (170.3)	1144.0 (162.7)	1114.1 (169.8)
Superior occipital gyrus (O1)	1131.5 (166.2)	1101.1 (167.7)	1251.1 (177.8)	1239.6 (186.7)
Posterior transverse collateral sulcus	300.7 (70.9)	294.2 (66.4)	373.0 (98.8)	386.9 (98.8)
Superior frontal sulcus	2004.9 (286.8)	2077.1 (302.7)	1867.6 (271.7)	1906.7 (296.1)
Sulcus intermedius primus (of Jensen)	280.2 (145.6)	257.6 (127.3)	350.1 (150.3)	364.0 (151.7)
Parieto-occipital sulcus (or fissure)	1445.9 (225.9)	1429.0 (239.3)	1584.5 (265.8)	1544.2 (255.8)

## Discussion

In a large sample of primarily young adult and healthy individuals, we tested for associations of handedness with total and regional measures of hemispheric cerebral cortical surface area. We report on the largest sample to have been analyzed to date in relation to this question. The proportion of left-handers in our sample was lower than in the general population, due to an exclusion of left-handers from some of the smaller studies that were pooled to create our BIG dataset. This exclusion bias, however, did not affect the heterogeneity of scan parameters present in both handedness groups, as reflected in the similar usage of headcoils between them. Nonetheless, we observed a sex difference in the incidence of left-handedness that was consistent with previous literature (with left-handedness occurring at an elevated rate in males; Sommer et al., 2008).

We did not observe any difference in bilateral cortical surface area in left-handers compared to right-handers. Nor did we find significant evidence for associations of handedness with region-specific bilateral surface areas, or their asymmetries, for regions related to language, hand motor control, or visual processing (Foundas et al., 1998, 1995; Willems et al., 2010). Our data therefore, provide little support for previously reported region-specific associations with handedness, although the Destrieux atlas' definitions of regions might not be identical to the definitions used in these previous studies. For example, the PT in the Destrieux atlas extends parietally (Destrieux et al., 2010), which is not a classic neuroanatomical definition (Geschwind and Levitsky, 1968; Steinmetz et al., 1991).

A limitation of our study was that, due to our large sample size and the number of cortical regions analyzed, systematic manual checking and adjustment of the automated parcellations was not feasible. Visual checks were made for only a small minority of images and not targeted to specific regions. However we exploited our subset of twice-scanned subjects in order to exclude regions that were not consistently parcellated from scan to re-scan, and also used outlier exclusion, as two forms of quality control. Clearly there is a need for improved methods of automated parcellation that capture some of the more variable and anatomically complex cortical regions better, in order to carry out future studies based on thousands of images. Another caveat is that the left and right definitions of cortical regions can only be considered “homologous” on the basis of information that was used in constructing the Destrieux atlas (that included information on cytoarchitecture), but this does not necessarily imply strict homology in genetic/developmental terms.

We found a suggestive association of handedness with the bilateral surface area of the superior part of the precentral sulcus, a region overlapping primary motor cortex. However, this association did not survive correction for multiple testing. Left-handers showed reduced surface areas compared to right handers in our sample (Table 4), which is at least consistent with the findings reported by Amunts et al. (1996) and Foundas et al. (1998). Males tend to have larger brains than females, which was also the case in our dataset, but this observed trend of decreased cerebral cortical surface area in left-handers was independent of this sex effect, and in the opposite direction to what might be predicted by it. Another suggestive association was found bilaterally with the inferior temporal gyrus. Again, left-handers in our sample showed reduced surface areas bilaterally (Table 4).

Our broader screen of non-candidate regional surface area and asymmetry differences between left- and right-handers did not identify significant novel associations. While relatively large, our sample size allowed us to detect standardized effect sizes regarded as medium (http://imaging.mrc-cbu.cam.ac.uk/statswiki/FAQ/effectSize), both before and after adjustment for multiple comparisons.

Although our dataset included a degree of heterogeneity in terms of scanning parameters used, there was no systematic difference in parameters applied for left- and right-handers, and we only analyzed measurements that showed a high scan–rescan correlation in twice-scanned subjects, despite this heterogeneity. Future studies based on even larger datasets will likely be affected by the same issue of heterogeneity, since large datasets are typically achieved through data pooling from multiple sources. It is therefore, encouraging that most of our measurements showed high scan–rescan correlations regardless of scanning heterogeneity.

An important issue in research on handedness is how exactly to define the trait. Many approaches have been taken to measure hand preference, ranging from motor performance measurements (e.g., relative hand skill, relative grip-strength; see Clerke and Clerke, 2001, for a brief overview); to self-report inventories assessing hand choice across various manual activities (Crovitz and Zener, 1962; Annett, 1967; Oldfield, 1971). Handedness inventories that account for preference across a range of tasks yield a rich assessment of (the degree of) handedness, and a detailed picture of its inter-subject variability. However, the resulting data are usually bimodal and are often subsequently dichotomized. For example, (Tan, 1993) showed that hand preference, when assessed by a very detailed questionnaire (Waterloo handedness questionnaire; Steenhuis and Bryden, 1989), shows a clear distinction between left-handed and right-handed populations. Further evidence for an intrinsic dichotomy in handedness was also provided by McManus (1991) who observed the same proportion of left-handers regardless of the questionnaire used. Accordingly, simple self-assessments of overall handedness, such as that used in the present study (asking subjects only to categorize themselves as left- or right-handed) show close agreement with dichotomous scoring of handedness as derived from multi-item inventories, as well as robust test–retest repeatability (Bryden et al., 1991; Tan, 1993; Ransil and Schachter, 1994). We are therefore confident of the validity of the binary, self-reported assessment of handedness that was used in our study.

Identifying cortical regional correlates of handedness may prove particularly useful in providing endophenotypes for future genetic studies of this trait, as well as clarifying the relationships between this and other forms of cerebral lateralization (Ocklenburg et al., 2013; Willems et al., 2014). We note that an association between handedness and cerebral cortical anatomy does not necessarily imply a simple causative relationship between the two. While it is conceivable that hand preference may arise due to hemispheric differences in cortical anatomy and function, it is equally conceivable that hand preference exerts developmental effects on cerebral cortical anatomy and function. As noted in the Introduction, there is strong evidence indicating that motor asymmetry of the arms and hands is initiated very early during human embryonic development, possibly even before the cerebral cortex exerts significant influence (Hepper, 2013). These early motor asymmetries, potentially under spino-muscular control, could therefore contribute to the determination of both handedness and regional cortical development.

Left-handed people show increased rates of reductions or reversals of lateralized brain functions, compared to right-handers (reviewed by Willems et al., 2014). Functional imaging studies of left-handers allow the possibility to study not only basic lateralization of brain function (e.g., of face perception), but also embodied cognition, and the extent of co-lateralization of different cognitive functions (Willems et al., 2014). Our survey of cerebral anatomical correlates of handedness may serve to inform these investigations, as it can suggest a prioritization of specific regions and cognitive processes to focus on with functional imaging techniques.

It is clear from our results, and those of previous studies, that any changes in brain structure associated with left-handedness are subtle. As noted earlier, it is likely that the genetic contributions to left-handedness are heterogeneous in nature, with multiple different genes being involved, and the same may be true of environmental influences (which also remain poorly understood). Etiologic heterogeneity suggests that there will be different forms of left-handedness which may manifest differently in terms of how striking any brain structural and functional correlates may be, and also differently in how, and to what extent, other lateralized cognitive systems are re-organized. A promising approach for studying the relations between lateralization and cognition will therefore be to specifically recruit left-handers, in order to recruit sufficient numbers for characterizing their heterogeneity, followed by assessments of brain structure and function in addition to neuropsychological testing, and genetic analysis (Marie et al., 2013; Mellet et al., 2013).

### Conflict of interest statement

The authors declare that the research was conducted in the absence of any commercial or financial relationships that could be construed as a potential conflict of interest.
