# Supplementary material for: Differences in cerebral cortical anatomy of left- and right-handers
Source: Front Psychol. 2014 Mar 28;5:261. doi: 10.3389/fpsyg.2014.00261 (PMC3975119; doi:10.3389/fpsyg.2014.00261)
Supplement: Supplementary file 1 [file DataSheet1.DOCX]

Supplementary Table 1. Repeated-measures ANOVA results from testing for an association between handedness and all regional cortical surface areas.

|  |  | **Repeated-measures ANOVA** | | |
| --- | --- | --- | --- | --- |
| **Region** |  | P | F | partial η2 |
| Inferior occipital gyrus (O3) and sulcus | Hand | 0.19 | 1.70 | <0.001 |
|  | Hand*hemisphere | 0.40 | 0.71 | <0.001 |
|  | Age | 0.79 | 0.07 | <0.001 |
|  | sex | 0.50 | 0.46 | <0.001 |
|  | TBS | 0.00 | 627.50 | 0.2344 |
|  | Scanner | 0.79 | 0.07 | <0.001 |
| Subcentral gyrus (central operculum) and sulci | Hand | 0.80 | 0.06 | <0.001 |
|  | Hand*hemisphere | 0.51 | 0.43 | <0.001 |
|  | Age | 0.39 | 0.72 | <0.001 |
|  | sex | 0.05 | 3.77 | 0.0018 |
|  | TBS | 0.00 | 1192.80 | 0.3682 |
|  | Scanner | 0.27 | 1.21 | <0.001 |
| Anterior part of the cingulate gyrus and sulcus (ACC) | Hand | 0.14 | 2.19 | 0.0011 |
|  | Hand*hemisphere | 0.02 | 5.18 | 0.0025 |
|  | Age | 0.53 | 0.40 | <0.001 |
|  | sex | 0.00 | 49.72 | 0.0236 |
|  | TBS | 0.00 | 3313.52 | 0.6172 |
|  | Scanner | 0.03 | 4.72 | 0.0023 |
| Middle-anterior part of the cingulate gyrus and sulcus (aMCC) | Hand | 0.67 | 0.18 | <0.001 |
|  | Hand*hemisphere | 0.00 | 8.99 | 0.0044 |
|  | Age | 0.86 | 0.03 | <0.001 |
|  | sex | 0.00 | 61.10 | 0.0289 |
|  | TBS | 0.00 | 2147.33 | 0.5115 |
|  | Scanner | 0.00 | 27.41 | 0.0132 |
| Middle-posterior part of the cingulate gyrus and sulcus (pMCC) | Hand | 0.54 | 0.37 | <0.001 |
|  | Hand*hemisphere | 0.98 | 0.00 | <0.001 |
|  | Age | 0.04 | 4.38 | 0.0021 |
|  | sex | 0.00 | 33.94 | 0.0163 |
|  | TBS | 0.00 | 2055.81 | 0.5007 |
|  | Scanner | 0.00 | 24.06 | 0.0116 |
| Posterior-dorsal part of the cingulate gyrus (dPCC) | Hand | 0.85 | 0.04 | <0.001 |
|  | Hand*hemisphere | 0.70 | 0.15 | <0.001 |
|  | Age | 0.83 | 0.04 | <0.001 |
|  | sex | 0.10 | 2.77 | 0.0014 |
|  | TBS | 0.00 | 1439.66 | 0.4131 |
|  | Scanner | 0.48 | 0.51 | <0.001 |
| Opercular part of the inferior frontal gyrus | Hand | 0.73 | 0.12 | <0.001 |
|  | Hand*hemisphere | 0.63 | 0.23 | <0.001 |
|  | Age | 0.46 | 0.54 | <0.001 |
|  | sex | 0.37 | 0.81 | <0.001 |
|  | TBS | 0.00 | 855.75 | 0.2953 |
|  | Scanner | 0.15 | 2.03 | 0.0010 |
| Triangular part of the inferior frontal gyrus | Hand | 0.88 | 0.02 | <0.001 |
|  | Hand*hemisphere | 0.17 | 1.84 | <0.001 |
|  | Age | 0.00 | 12.72 | 0.0062 |
|  | sex | 0.04 | 4.21 | 0.0020 |
|  | TBS | 0.00 | 345.28 | 0.1441 |
|  | Scanner | 0.79 | 0.07 | <0.001 |
| Middle frontal gyrus (F2) | Hand | 0.88 | 0.02 | <0.001 |
|  | Hand*hemisphere | 0.88 | 0.02 | <0.001 |
|  | Age | 0.01 | 6.30 | 0.0031 |
|  | sex | 0.00 | 35.94 | 0.0172 |
|  | TBS | 0.00 | 2583.83 | 0.5571 |
|  | Scanner | 0.84 | 0.04 | <0.001 |
| Superior frontal gyrus (F1) | Hand | 0.66 | 0.19 | <0.001 |
|  | Hand*hemisphere | 0.31 | 1.03 | <0.001 |
|  | Age | 0.00 | 10.67 | 0.0052 |
|  | sex | 0.05 | 3.73 | 0.0018 |
|  | TBS | 0.00 | 3304.86 | 0.6166 |
|  | Scanner | 0.53 | 0.39 | <0.001 |
| Middle occipital gyrus (O2, lateral occipital gyrus) | Hand | 0.75 | 0.10 | <0.001 |
|  | Hand*hemisphere | 0.35 | 0.86 | <0.001 |
|  | Age | 0.98 | 0.00 | <0.001 |
|  | sex | 0.26 | 1.25 | <0.001 |
|  | TBS | 0.00 | 1471.02 | 0.4177 |
|  | Scanner | 0.00 | 12.96 | 0.0063 |
| Superior occipital gyrus (O1) | Hand | 0.04 | 4.24 | 0.0021 |
|  | Hand*hemisphere | 0.25 | 1.30 | <0.001 |
|  | Age | 0.01 | 6.07 | 0.0030 |
|  | sex | 0.00 | 14.51 | 0.0070 |
|  | TBS | 0.00 | 761.37 | 0.2713 |
|  | Scanner | 0.36 | 0.84 | <0.001 |
| Lateral occipito-temporal gyrus (fusiform gyrus, O4-T4) | Hand | 0.17 | 1.87 | <0.001 |
|  | Hand*hemisphere | 0.53 | 0.40 | <0.001 |
|  | Age | 0.04 | 4.06 | 0.0020 |
|  | sex | 0.03 | 4.69 | 0.0023 |
|  | TBS | 0.00 | 701.18 | 0.2545 |
|  | Scanner | 0.20 | 1.63 | <0.001 |
| Lingual gyrus, ligual part of the medial occipito-temporal gyrus, (O5) | Hand | 0.26 | 1.27 | <0.001 |
|  | Hand*hemisphere | 0.10 | 2.67 | 0.0013 |
|  | Age | 0.18 | 1.77 | <0.001 |
|  | sex | 0.43 | 0.63 | <0.001 |
|  | TBS | 0.00 | 944.86 | 0.3156 |
|  | Scanner | 0.00 | 19.91 | 0.0096 |
| Angular gyrus | Hand | 0.94 | 0.01 | <0.001 |
|  | Hand*hemisphere | 0.77 | 0.09 | <0.001 |
|  | Age | 0.13 | 2.34 | 0.0011 |
|  | sex | 0.04 | 4.23 | 0.0021 |
|  | TBS | 0.00 | 857.14 | 0.2947 |
|  | Scanner | 0.77 | 0.08 | <0.001 |
| Supramarginal gyrus | Hand | 0.20 | 1.66 | <0.001 |
|  | Hand*hemisphere | 0.78 | 0.08 | <0.001 |
|  | Age | 0.00 | 9.68 | 0.0047 |
|  | sex | 0.36 | 0.84 | <0.001 |
|  | TBS | 0.00 | 1466.62 | 0.4171 |
|  | Scanner | 0.55 | 0.36 | <0.001 |
| Superior parietal lobule (lateral part of P1) | Hand | 0.65 | 0.21 | <0.001 |
|  | Hand*hemisphere | 0.81 | 0.06 | <0.001 |
|  | Age | 0.36 | 0.82 | <0.001 |
|  | sex | 0.04 | 4.15 | 0.0020 |
|  | TBS | 0.00 | 1090.63 | 0.3476 |
|  | Scanner | 0.23 | 1.42 | <0.001 |
| Precuneus (medial part of P1) | Hand | 0.17 | 1.88 | <0.001 |
|  | Hand*hemisphere | 0.78 | 0.08 | <0.001 |
|  | Age | 0.00 | 11.25 | 0.0055 |
|  | sex | 0.11 | 2.63 | 0.0013 |
|  | TBS | 0.00 | 1033.27 | 0.3358 |
|  | Scanner | 0.21 | 1.55 | <0.001 |
| Anterior transverse collateral sulcus | Hand | 0.86 | 0.03 | <0.001 |
|  | Hand*hemisphere | 0.06 | 3.44 | 0.0017 |
|  | Age | 0.32 | 1.00 | <0.001 |
|  | sex | 0.98 | 0.00 | <0.001 |
|  | TBS | 0.00 | 722.57 | 0.2603 |
|  | Scanner | 0.98 | 0.00 | <0.001 |
| Lateral aspect of the superior temporal gyrus | Hand | 0.57 | 0.33 | <0.001 |
|  | Hand*hemisphere | 0.36 | 0.85 | <0.001 |
|  | Age | 0.00 | 9.06 | 0.0044 |
|  | sex | 0.17 | 1.90 | <0.001 |
|  | TBS | 0.00 | 1634.83 | 0.4438 |
|  | Scanner | 0.00 | 9.82 | 0.0048 |
| Planum temporale or temporal plane of the superior temporal gyrus | Hand | 0.42 | 0.64 | <0.001 |
|  | Hand*hemisphere | 0.94 | 0.01 | <0.001 |
|  | Age | 0.63 | 0.23 | <0.001 |
|  | sex | 0.00 | 14.39 | 0.0070 |
|  | TBS | 0.00 | 753.83 | 0.2695 |
|  | Scanner | 0.91 | 0.01 | <0.001 |
| Inferior temporal gyrus (T3) | Hand | 0.04 | 4.36 | 0.0021 |
|  | Hand*hemisphere | 0.58 | 0.30 | <0.001 |
|  | Age | 0.50 | 0.46 | <0.001 |
|  | sex | 0.14 | 2.15 | 0.0010 |
|  | TBS | 0.00 | 1366.81 | 0.3994 |
|  | Scanner | 0.39 | 0.75 | <0.001 |
| Middle temporal gyrus (T2) | Hand | 0.61 | 0.27 | <0.001 |
|  | Hand*hemisphere | 0.91 | 0.01 | <0.001 |
|  | Age | 0.01 | 6.24 | 0.0030 |
|  | sex | 0.97 | 0.00 | <0.001 |
|  | TBS | 0.00 | 2080.20 | 0.5034 |
|  | Scanner | 0.55 | 0.35 | <0.001 |
| Horizontal ramus of the anterior segment of the lateral sulcus (or fissure) | Hand | 0.22 | 1.49 | <0.001 |
|  | Hand*hemisphere | 0.38 | 0.77 | <0.001 |
|  | Age | 0.21 | 1.58 | <0.001 |
|  | sex | 0.00 | 8.25 | 0.0040 |
|  | TBS | 0.00 | 247.20 | 0.1079 |
|  | Scanner | 0.59 | 0.29 | <0.001 |
| Vertical ramus of the anterior segment of the lateral sulcus (or fissure) | Hand | 0.77 | 0.08 | <0.001 |
|  | Hand*hemisphere | 0.76 | 0.09 | <0.001 |
|  | Age | 0.11 | 2.55 | 0.0012 |
|  | sex | 0.60 | 0.28 | <0.001 |
|  | TBS | 0.00 | 160.09 | 0.0725 |
|  | Scanner | 0.98 | 0.00 | <0.001 |
| Posterior ramus (or segment) of the lateral sulcus (or fissure) | Hand | 0.80 | 0.06 | <0.001 |
|  | Hand*hemisphere | 0.62 | 0.24 | <0.001 |
|  | Age | 0.45 | 0.57 | <0.001 |
|  | sex | 0.00 | 12.12 | 0.0059 |
|  | TBS | 0.00 | 605.27 | 0.2285 |
|  | Scanner | 0.51 | 0.44 | <0.001 |
| Calcarine sulcus | Hand | 0.16 | 1.99 | <0.001 |
|  | Hand*hemisphere | 0.12 | 2.36 | 0.0011 |
|  | Age | 0.01 | 7.31 | 0.0035 |
|  | sex | 0.00 | 8.37 | 0.0041 |
|  | TBS | 0.00 | 814.71 | 0.2839 |
|  | Scanner | 0.00 | 8.45 | 0.0041 |
| Anterior segment of the circular sulcus of the insula | Hand | 0.61 | 0.26 | <0.001 |
|  | Hand*hemisphere | 0.91 | 0.01 | <0.001 |
|  | Age | 0.20 | 1.65 | <0.001 |
|  | sex | 0.01 | 6.31 | 0.0031 |
|  | TBS | 0.00 | 522.18 | 0.2031 |
|  | Scanner | 0.62 | 0.24 | <0.001 |
| Superior segment of the circular sulcus of the insula | Hand | 0.38 | 0.79 | <0.001 |
|  | Hand*hemisphere | 0.66 | 0.19 | <0.001 |
|  | Age | 0.21 | 1.55 | <0.001 |
|  | sex | 0.00 | 9.02 | 0.0044 |
|  | TBS | 0.00 | 1215.05 | 0.3719 |
|  | Scanner | 0.00 | 9.06 | 0.0044 |
| Anterior transverse collateral sulcus | Hand | 0.85 | 0.04 | <0.001 |
|  | Hand*hemisphere | 0.56 | 0.35 | <0.001 |
|  | Age | 0.00 | 8.43 | 0.0041 |
|  | sex | 0.00 | 12.39 | 0.0060 |
|  | TBS | 0.00 | 448.10 | 0.1792 |
|  | Scanner | 0.19 | 1.70 | <0.001 |
| Posterior transverse collateral sulcus | Hand | 0.65 | 0.21 | <0.001 |
|  | Hand*hemisphere | 0.05 | 3.92 | 0.0019 |
|  | Age | 0.13 | 2.30 | 0.0011 |
|  | sex | 0.04 | 4.23 | 0.0021 |
|  | TBS | 0.00 | 181.55 | 0.0812 |
|  | Scanner | 0.05 | 3.95 | 0.0019 |
| Inferior frontal sulcus | Hand | 0.62 | 0.25 | <0.001 |
|  | Hand*hemisphere | 0.57 | 0.31 | <0.001 |
|  | Age | 0.04 | 4.15 | 0.0020 |
|  | sex | 0.00 | 29.75 | 0.0143 |
|  | TBS | 0.00 | 1546.64 | 0.4299 |
|  | Scanner | 0.55 | 0.36 | <0.001 |
| Middle frontal sulcus | Hand | 0.78 | 0.08 | <0.001 |
|  | Hand*hemisphere | 0.61 | 0.26 | <0.001 |
|  | Age | 0.67 | 0.18 | <0.001 |
|  | sex | 0.30 | 1.07 | <0.001 |
|  | TBS | 0.00 | 896.02 | 0.3037 |
|  | Scanner | 0.24 | 1.38 | <0.001 |
| Superior frontal sulcus | Hand | 0.04 | 4.31 | 0.0021 |
|  | Hand*hemisphere | 0.22 | 1.50 | <0.001 |
|  | Age | 0.00 | 15.57 | 0.0075 |
|  | sex | 0.00 | 12.82 | 0.0062 |
|  | TBS | 0.00 | 1491.50 | 0.4208 |
|  | Scanner | 0.64 | 0.22 | <0.001 |
| Sulcus intermedius primus (of Jensen) | Hand | 0.71 | 0.14 | <0.001 |
|  | Hand*hemisphere | 0.04 | 4.37 | 0.0021 |
|  | Age | 0.64 | 0.22 | <0.001 |
|  | sex | 0.00 | 12.28 | 0.0060 |
|  | TBS | 0.00 | 86.72 | 0.0407 |
|  | Scanner | 0.58 | 0.31 | <0.001 |
| Intraparietal sulcus (interparietal sulcus) and transverse parietal sulci | Hand | 0.92 | 0.01 | <0.001 |
|  | Hand*hemisphere | 0.52 | 0.40 | <0.001 |
|  | Age | 0.32 | 1.00 | <0.001 |
|  | sex | 0.77 | 0.09 | <0.001 |
|  | TBS | 0.00 | 1088.35 | 0.3468 |
|  | Scanner | 0.64 | 0.22 | <0.001 |
| Middle occipital sulcus and lunatus sulcus | Hand | 0.71 | 0.14 | <0.001 |
|  | Hand*hemisphere | 0.75 | 0.11 | <0.001 |
|  | Age | 0.30 | 1.07 | <0.001 |
|  | sex | 0.04 | 4.27 | 0.0021 |
|  | TBS | 0.00 | 474.74 | 0.1877 |
|  | Scanner | 0.00 | 15.57 | 0.0075 |
| Superior occipital sulcus and transverse occipital sulcus | Hand | 0.27 | 1.21 | <0.001 |
|  | Hand*hemisphere | 0.81 | 0.06 | <0.001 |
|  | Age | 0.76 | 0.09 | <0.001 |
|  | sex | 0.60 | 0.28 | <0.001 |
|  | TBS | 0.00 | 701.28 | 0.2544 |
|  | Scanner | 0.01 | 7.05 | 0.0034 |
| Anterior occipital sulcus and preoccipital notch (temporo-occipital incisure) | Hand | 0.78 | 0.08 | <0.001 |
|  | Hand*hemisphere | 0.68 | 0.17 | <0.001 |
|  | Age | 0.01 | 6.69 | 0.0033 |
|  | sex | 0.85 | 0.03 | <0.001 |
|  | TBS | 0.00 | 246.08 | 0.1072 |
|  | Scanner | 0.39 | 0.75 | <0.001 |
| Lateral occipito-temporal sulcus | Hand | 0.35 | 0.86 | <0.001 |
|  | Hand*hemisphere | 0.91 | 0.01 | <0.001 |
|  | Age | 0.00 | 13.32 | 0.0064 |
|  | sex | 0.00 | 17.07 | 0.0082 |
|  | TBS | 0.00 | 573.48 | 0.2182 |
|  | Scanner | 0.55 | 0.35 | <0.001 |
| Medial occipito-temporal sulcus (collateral sulcus) and lingual sulcus | Hand | 0.96 | 0.00 | <0.001 |
|  | Hand*hemisphere | 0.15 | 2.06 | 0.0010 |
|  | Age | 0.02 | 5.28 | 0.0026 |
|  | sex | 0.00 | 24.28 | 0.0117 |
|  | TBS | 0.00 | 641.15 | 0.2382 |
|  | Scanner | 0.00 | 15.86 | 0.0077 |
| Lateral orbital sulcus | Hand | 0.79 | 0.07 | <0.001 |
|  | Hand*hemisphere | 0.73 | 0.12 | <0.001 |
|  | Age | 0.13 | 2.30 | 0.0011 |
|  | sex | 0.09 | 2.82 | 0.0014 |
|  | TBS | 0.00 | 456.96 | 0.1823 |
|  | Scanner | 0.11 | 2.52 | 0.0012 |
| Parieto-occipital sulcus (or fissure) | Hand | 0.03 | 4.80 | 0.0023 |
|  | Hand*hemisphere | 0.25 | 1.32 | <0.001 |
|  | Age | 0.48 | 0.49 | <0.001 |
|  | sex | 0.33 | 0.95 | <0.001 |
|  | TBS | 0.00 | 868.76 | 0.2975 |
|  | Scanner | 0.00 | 8.90 | 0.0043 |
| Pericallosal sulcus (S of corpus callosum) | Hand | 0.57 | 0.32 | <0.001 |
|  | Hand*hemisphere | 0.84 | 0.04 | <0.001 |
|  | Age | 0.00 | 18.26 | 0.0088 |
|  | sex | 0.81 | 0.06 | <0.001 |
|  | TBS | 0.00 | 1124.37 | 0.3544 |
|  | Scanner | 0.00 | 12.17 | 0.0059 |
| Postcentral sulcus | Hand | 0.45 | 0.56 | <0.001 |
|  | Hand*hemisphere | 0.77 | 0.08 | <0.001 |
|  | Age | 0.00 | 15.44 | 0.0075 |
|  | sex | 0.25 | 1.34 | <0.001 |
|  | TBS | 0.00 | 1091.54 | 0.3476 |
|  | Scanner | 0.56 | 0.33 | <0.001 |
| Inferior part of the precentral sulcus | Hand | 0.76 | 0.09 | <0.001 |
|  | Hand*hemisphere | 0.85 | 0.04 | <0.001 |
|  | Age | 0.42 | 0.65 | <0.001 |
|  | sex | 0.00 | 12.26 | 0.0059 |
|  | TBS | 0.00 | 790.43 | 0.2783 |
|  | Scanner | 0.18 | 1.78 | <0.001 |
| Superior part of the precentral sulcus | Hand | 0.04 | 4.07 | 0.0020 |
|  | Hand*hemisphere | 0.60 | 0.28 | <0.001 |
|  | Age | 0.08 | 2.99 | 0.0015 |
|  | sex | 0.00 | 21.10 | 0.0102 |
|  | TBS | 0.00 | 396.44 | 0.1625 |
|  | Scanner | 0.37 | 0.82 | <0.001 |
| Subparietal sulcus | Hand | 0.07 | 3.40 | 0.0017 |
|  | Hand*hemisphere | 0.21 | 1.59 | <0.001 |
|  | Age | 0.00 | 8.62 | 0.0042 |
|  | sex | 0.67 | 0.18 | <0.001 |
|  | TBS | 0.00 | 583.58 | 0.2221 |
|  | Scanner | 0.00 | 9.44 | 0.0046 |
| Inferior temporal sulcus | Hand | 0.52 | 0.42 | <0.001 |
|  | Hand*hemisphere | 0.40 | 0.71 | <0.001 |
|  | Age | 0.03 | 4.90 | 0.0024 |
|  | sex | 0.03 | 4.71 | 0.0023 |
|  | TBS | 0.00 | 914.62 | 0.3080 |
|  | Scanner | 0.00 | 16.09 | 0.0078 |
| Superior temporal sulcus (parallel sulcus) | Hand | 0.45 | 0.56 | <0.001 |
|  | Hand*hemisphere | 0.90 | 0.02 | <0.001 |
|  | Age | 0.04 | 4.42 | 0.0022 |
|  | sex | 0.09 | 2.94 | 0.0014 |
|  | TBS | 0.00 | 1737.24 | 0.4588 |
|  | Scanner | 0.93 | 0.01 | <0.001 |
| RightS_temporal_transverse Transverse temporal sulcus | Hand | 0.84 | 0.04 | <0.001 |
|  | Hand*hemisphere | 0.45 | 0.57 | <0.001 |
|  | Age | 0.82 | 0.05 | <0.001 |
|  | sex | 0.42 | 0.64 | <0.001 |
|  | TBS | 0.00 | 439.54 | 0.1766 |
|  | Scanner | 0.44 | 0.60 | <0.001 |

Supplementary Table 2. Means and SDs for all cortical regions, by hemisphere and handedness group.

|  | left hemisphere | | | | right hemisphere | | | | |
| --- | --- | --- | --- | --- | --- | --- | --- | --- | --- |
|  | righthanders | | lefthanders | | righthanders | | lefthanders | | |
|  | Mean | Standard Deviation | Mean | Standard Deviation | Mean | Standard Deviation | Mean | Standard Deviation | |
| Inferior occipital gyrus (O3) and sulcus | 1137.2 | 202.79 | 1162.34 | 206.42 | 921.82 | 178.69 | 924.04 | | 160.17 |
| Subcentral gyrus (central operculum) and sulci | 1039.72 | 169.11 | 1027.19 | 151.92 | 946.8 | 161.05 | 944.39 | | 156.9 |
| Anterior part of the cingulate gyrus and sulcus (ACC) | 1707.41 | 264.52 | 1648 | 223.25 | 2016.75 | 271.05 | 1998.77 | | 251.29 |
| Middle-anterior part of the cingulate gyrus and sulcus (aMCC) | 1014.45 | 170.26 | 974.63 | 144.66 | 1114.09 | 169.76 | 1122.02 | | 162.75 |
| Middle-posterior part of the cingulate gyrus and sulcus (pMCC) | 945.9 | 126.09 | 934.8 | 128.56 | 1043.41 | 155.18 | 1026.1 | | 135.02 |
| Posterior-dorsal part of the cingulate gyrus (dPCC) | 397.35 | 87.3 | 393.2 | 93.02 | 375.4 | 81.73 | 374.67 | | 80.68 |
| Opercular part of the inferior frontal gyrus | 1014.87 | 162.05 | 999.85 | 142.84 | 900.96 | 154.42 | 896.73 | | 135.3 |
| Triangular part of the inferior frontal gyrus | 800.31 | 150.68 | 809.84 | 152.82 | 785.73 | 170.98 | 773.11 | | 175.4 |
| Middle frontal gyrus (F2) | 3211.03 | 507.53 | 3183.17 | 467.43 | 2927.62 | 472.32 | 2897.2 | | 482.66 |
| Superior frontal gyrus (F1) | 5013.14 | 603.73 | 4986.29 | 555.95 | 4749.46 | 567.49 | 4687.52 | | 537.41 |
| Middle occipital gyrus (O2, lateral occipital gyrus) | 1480.77 | 246.44 | 1463.92 | 235.04 | 1587.71 | 267.51 | 1598.36 | | 272.15 |
| Superior occipital gyrus (O1) | 1101.09 | 167.73 | 1131.45 | 166.24 | 1239.58 | 186.67 | 1251.14 | | 177.79 |
| Lateral occipito-temporal gyrus (fusiform gyrus, O4-T4) | 1348.41 | 241.17 | 1372.33 | 248.25 | 1311.04 | 248.93 | 1320.72 | | 261.33 |
| Lingual gyrus, ligual part of the medial occipito-temporal gyrus, (O5) | 2103 | 328.95 | 2142.25 | 356.76 | 2008.1 | 286.43 | 1996.52 | | 325.12 |
| Angular gyrus | 1714.58 | 278.22 | 1701.25 | 282.77 | 2074.69 | 344.06 | 2067.43 | | 340.04 |
| Supramarginal gyrus | 2103.44 | 340.95 | 2127.71 | 352.52 | 1924.38 | 307.98 | 1930.42 | | 272.81 |
| lh_G_parietal_sup_area | 2091.5 | 328.85 | 2088.86 | 343.75 | 1712.15 | 279.01 | 1707.27 | | 294.14 |
| lh_G_precuneus_area | 1863.42 | 287.73 | 1828.92 | 287.89 | 1849.88 | 278.47 | 1815.72 | | 248.13 |
| Superior parietal lobule (lateral part of P1) | 358.77 | 78.14 | 349.34 | 70.48 | 278.14 | 58.92 | 281.85 | | 59.26 |
| Lateral aspect of the superior temporal gyrus | 1456.86 | 186.05 | 1466.91 | 175.07 | 1274.63 | 168.94 | 1267.28 | | 162.88 |
| Planum temporale or temporal plane of the superior temporal gyrus | 683.35 | 154.87 | 691.1 | 157.56 | 555.83 | 109.3 | 559.35 | | 106.24 |
| Inferior temporal gyrus (T3) | 1911.66 | 311.34 | 1853.15 | 328.58 | 1787.62 | 281.76 | 1744.8 | | 319.6 |
| Middle temporal gyrus (T2) | 2029.02 | 300.93 | 2024.9 | 310.95 | 2128.28 | 291.58 | 2133.33 | | 324.04 |
| Horizontal ramus of the anterior segment of the lateral sulcus (or fissure) | 234.27 | 43.37 | 232.04 | 37.38 | 288.28 | 55.88 | 281.03 | | 56.46 |
| Vertical ramus of the anterior segment of the lateral sulcus (or fissure) | 208.72 | 60.82 | 205.89 | 61.66 | 154.22 | 46.92 | 153.2 | | 48.08 |
| Posterior ramus (or segment) of the lateral sulcus (or fissure) | 839.61 | 136.54 | 838.57 | 134.45 | 995.48 | 110.39 | 998.1 | | 104.69 |
| Calcarine sulcus | 1763.54 | 308.79 | 1797.71 | 324.43 | 1691.94 | 296.65 | 1696.94 | | 303.44 |
| Anterior segment of the circular sulcus of the insula | 372.02 | 63.86 | 373.01 | 64.04 | 425.19 | 79.96 | 427.32 | | 72.91 |
| Superior segment of the circular sulcus of the insula | 1259.87 | 134.4 | 1250.75 | 133.62 | 986.18 | 121.1 | 974.08 | | 121.06 |
| Anterior transverse collateral sulcus | 713.82 | 182.13 | 698.27 | 179.42 | 737.34 | 165.56 | 729.97 | | 168.17 |
| Posterior transverse collateral sulcus | 294.23 | 66.4 | 300.74 | 70.88 | 386.9 | 98.8 | 373.01 | | 98.78 |
| Inferior frontal sulcus | 1679.15 | 284.16 | 1663.48 | 256.61 | 1543.08 | 256.64 | 1509.46 | | 263.13 |
| Middle frontal sulcus | 1136.41 | 230.89 | 1117.36 | 232.76 | 1576.87 | 301.52 | 1566.79 | | 276.07 |
| Superior frontal sulcus | 2077.1 | 302.72 | 2004.92 | 286.84 | 1906.66 | 296.12 | 1867.58 | | 271.67 |
| Sulcus intermedius primus (of Jensen) | 257.6 | 127.33 | 280.16 | 145.57 | 363.98 | 151.67 | 350.14 | | 150.34 |
| Intraparietal sulcus (interparietal sulcus) and transverse parietal sulci | 2201.93 | 314.74 | 2195.7 | 357.47 | 2289.58 | 333.47 | 2275.45 | | 337.06 |
| Middle occipital sulcus and lunatus sulcus | 803.55 | 192.92 | 797.9 | 197.2 | 745.37 | 195.68 | 732.94 | | 203.21 |
| Superior occipital sulcus and transverse occipital sulcus | 907.87 | 168.6 | 913.75 | 159.34 | 1077.04 | 208.71 | 1089.03 | | 199.94 |
| Anterior occipital sulcus and preoccipital notch (temporo-occipital incisure) | 575.01 | 152.98 | 571.27 | 154.42 | 557.58 | 145.81 | 560.32 | | 155.92 |
| Lateral occipito-temporal sulcus | 682.69 | 156.2 | 668.5 | 152.16 | 713.25 | 158.52 | 702.25 | | 176.95 |
| Medial occipito-temporal sulcus (collateral sulcus) and lingual sulcus | 1483.23 | 243.14 | 1497.21 | 250.24 | 1380.02 | 210.86 | 1369.28 | | 218.22 |
| Lateral orbital sulcus | 288.07 | 64.9 | 287.16 | 70 | 324.7 | 81.61 | 325.39 | | 77.07 |
| Parieto-occipital sulcus (or fissure) | 1428.97 | 239.29 | 1445.91 | 225.88 | 1544.17 | 255.76 | 1584.46 | | 265.83 |
| Pericallosal sulcus (S of corpus callosum) | 782.94 | 147.05 | 773.28 | 139.56 | 1046.12 | 187.98 | 1033.8 | | 173.13 |
| Postcentral sulcus | 2111.17 | 343.64 | 2113.72 | 367.47 | 1786.79 | 330.98 | 1795.69 | | 368.98 |
| Inferior part of the precentral sulcus | 1079.92 | 193.18 | 1074.39 | 212.45 | 1181.15 | 203.31 | 1178.31 | | 182.64 |
| Superior part of the precentral sulcus | 952.67 | 200.9 | 914.87 | 207.54 | 990.44 | 214.78 | 965.08 | | 201.89 |
| Subparietal sulcus | 790.95 | 175.17 | 768.86 | 168.68 | 881.42 | 208.55 | 838.87 | | 206.23 |
| Inferior temporal sulcus | 987.2 | 232.3 | 976.31 | 243.09 | 921.54 | 211.05 | 893.53 | | 214.27 |
| Superior temporal sulcus (parallel sulcus) | 3955.2 | 500.88 | 3922.28 | 501.44 | 4353.06 | 559.69 | 4311.56 | | 639.29 |
| Transverse temporal sulcus | 265.52 | 52.76 | 261.74 | 42.2 | 213.6 | 48.17 | 214.58 | | 43.68 |
